# Day-Ahead Hourly Solar Irradiance Forecasting Based on Multi-Attributed Spatio-Temporal Graph Convolutional Network

**DOI:** 10.3390/s22197179

**Published:** 2022-09-21

**Authors:** Hyeon-Ju Jeon, Min-Woo Choi, O-Joun Lee

**Affiliations:** 1 Data Assimilation Group, Korea Institute of Atmospheric Prediction Systems (KIAPS), 35, Boramae-ro 5-gil, Dongjak-gu, Seoul 07059, Korea; 2 Department of Artificial Intelligence, The Catholic University of Korea, 43, Jibong-ro, Bucheon-si 14662, Korea

**Keywords:** solar irradiance forecasting, graph neural network, spatio-temporal graph convolutional network, multivariate spatio-temporal analysis, weather forecasting

## Abstract

Solar irradiance forecasting is fundamental and essential for commercializing solar energy generation by overcoming output variability. Accurate forecasting depends on historical solar irradiance data, correlations between various meteorological variables (e.g., wind speed, humidity, and cloudiness), and influences between the weather contexts of spatially adjacent regions. However, existing studies have been limited to spatiotemporal analysis of a few variables, which have clear correlations with solar irradiance (e.g., sunshine duration), and do not attempt to establish atmospheric contextual information from a variety of meteorological variables. Therefore, this study proposes a novel solar irradiance forecasting model that represents atmospheric parameters observed from multiple stations as an attributed dynamic network and analyzes temporal changes in the network by extending existing spatio-temporal graph convolutional network (ST-GCN) models. By comparing the proposed model with existing models, we also investigated the contributions of (i) the spatial adjacency of the stations, (ii) temporal changes in the meteorological variables, and (iii) the variety of variables to the forecasting performance. We evaluated the performance of the proposed and existing models by predicting the hourly solar irradiance at observation stations in the Korean Peninsula. The experimental results showed that the three features are synergistic and have correlations that are difficult to establish using single-aspect analysis.

## 1. Introduction

Extensive growth in the global population has led to an increase in the use of fossil fuels and greenhouse gas emissions, leading to worsening environmental pollution and global warming problems [1]. In 2015, the United States and China pledged to achieve 100% reliance on renewable energy to tackle climate change [2]. In addition, the European Union has decided to reduce greenhouse gas emissions and transition to renewable energy entirely by 2050 [3]. Among renewable energy sources, solar (photovoltaic) energy is estimated to meet a quarter of the electricity demand by 2050 [4]. However, because various factors, such as solar position, time, geographical location, and meteorological conditions, affect solar power generation, the efficiency of solar power plants is highly volatile [5,6]. Volatility also causes problems such as output instability of solar power plants and overloads of power grids, which should be addressed for commercializing solar energy [7,8,9,10]. Therefore, methods for accurately forecasting solar energy production in a specific region have become essential [11,12]. Various solar irradiance prediction models, from statistical to neural network-empowered, have been proposed for providing a scientific basis for managing solar power generation and power grid overloads [13,14,15,16].

Conventional solar irradiance forecasting models can be classified as physical, empirical, and statistical models. The physical approach represents meteorological conditions in a region with three-dimensional grids and model correlations between meteorological variables with nonlinear functions based on atmospheric physics [17,18]. However, physical models have extremely high computational and space complexity, and their performance is significantly affected by initial conditions, grid resolution, and data uncertainty [19,20,21]. The empirical approach, which is the most widely used, applies regression models for analyzing the correlations between various meteorological variables, such as sunshine duration, cloudiness, and temperature [22]. Empirical models are simple and intuitive, but their accuracy is insufficient for practical use [23,24,25]. The statistical approach predicts solar irradiance based on temporal correlations between historical meteorological variables using statistical models, such as the autoregressive moving average model (ARMA) [26] and autoregressive integrated moving average model (ARIMA) [27]. Although the statistical approach shows a higher performance than the empirical approach, it is challenging for both approaches to represent and explain non-linear correlations between the meteorological variables considering model interpretability [28]. In addition, both the empirical and statistical approaches have far lower computational and space complexities than the physical approach. However, their performance is simultaneously insufficient for replacing the physical approach based on atmospheric knowledge.

To improve the performance of the empirical and statistical approaches, machine learning (ML) models such as support vector machines (SVM) and artificial neural networks (ANN) have been highlighted as effective tools for representing complicated correlations between meteorological variables [29,30]. Lyra et al. [31] and Chen et al. [32] applied ANN and SVM to predict daily solar irradiance, respectively. In addition, Sun et al. [33] estimated daily solar irradiance in China using the random forest model. ML models follow an approach similar to that of statistical models but have greater expressive power and higher accuracy [34]. However, existing ML models remain insufficient for analyzing correlations between multiple meteorological variables collected from multiple observation stations and for providing solar irradiance forecasting with high time resolution [35].

Thus, recent studies have focused on deep-learning-based models that stack multiple neural network layers for improving the expressive power of forecasting models. Venugopal et al. [36] predicted the power output of a solar panel after 15 min by analyzing the power output for the past 15 min and the ground sky image using a convolutional neural network (CNN) model. Aslam et al. [37] conducted a comparative study of the annual solar irradiance forecasting performance of gated recurrent unit (GRU), long short-term memory (LSTM), multilayer perceptron (MLP), SVM, and random forest models. Heo et al. [38] applied a CNN model to a digital elevation map to extract topographic features that can be used as a reference for locating solar panels. Topographical features are effective in predicting the annual solar irradiance. The European Center for Intermediate-Range Weather Forecasting (ECMWF), a well-known organization with global weather forecasting capabilities, is also conducting research for improving forecasting performance by using deep learning-based models [39,40]. However, most of existing deep learning-empowered models depend on a single feature (i.e., temporal [36,37] or spatial feature [38]). Merely applying CNN to geospatial data fitted into grids and recurrent neural network (RNN) to time-sequential observations could not improve the model interpretability, not only underperforming atmospheric knowledge-based models.

As a sufficient number of spatiotemporal meteorological datasets have become available, hybrid neural network models, which aim to combine spatial and temporal features, have been highlighted for improving the practicality and accuracy of forecasting models [41]. Wang et al. [42] proposed a novel model that combines LSTM with a CNN for predicting solar power production. This model extracts the temporal features of each meteorological variable using LSTM layers and applies CNN layers to the temporal features to conduct spatial analysis. However, the observation stations were not located in square grids. In addition, the spatial adjacency of the stations does not always correspond to the spatial influences between the weather contexts of the stations. Thus, graphs and graph neural network (GNN) models are more effective in representing the nonuniform spatial adjacency and analyzing the influences than grids and CNN models, respectively. Jiao et al. [43] composed a graph with observation stations and their adjacencies. Adjacency was defined based on Pearson correlation coefficients (PCC) between the historical solar irradiance at each station and the minimum threshold. They then predicted future solar irradiance by analyzing the spatial influences of the GNN layers and applying LSTM layers to the spatial features extracted by the GNN layers. Khodayar and Wang [44] applied a similar approach to wind-speed forecasting. They defined spatial adjacency using the mutual information between historical wind speeds and directions. In contrast to Jiao et al. [43], Khodayar and Wang first extracted temporal features from each station using LSTM and then used the GNN for analyzing the spatial correlations between the temporal features. Dong et al. [45] used dilated CNN layers instead of RNN layers for analyzing the temporal characteristics of wind power production. Because CNN layers are more effective in parallelizing its computation than recurrent models, this approach could reduce the time consumption for both model training and forecasting services. However, CNN layers have limitations in explicitly considering the time-sequential characteristics of the meteorological data. Muthukumar et al. [46] predicted PM 2.5 concentration by combining graph convolutional network (GCN) and ConvLSTM and constructed a graph using the distance between fine dust sensors and interpolated data from unobserved locations using the GCN layer. The output of the GCN for spatial interpolation was converted into an image and input into ConvLSTM. Spatiotemporal models performed better than models without fusing spatial and temporal information [47,48]. However, these models cannot utilize a variety of meteorological data (e.g., humidity, temperature, air pressure, and cloudiness) observed with prediction targets. This makes the conventional models far from understanding the weather conditions of observation stations and their spatiotemporal correlations. Cheng et al. [49] used GNN to analyze correlations between atmospheric variables, and Huang et al. [50] extracted the temporal features of each variable and analyzed feature correlations using MLP. However, these studies omitted the spatial influences between observation stations.

The weather conditions of spatially adjacent observation stations influence each other; for example, clouds move with wind. The influences occur with non-uniform time lags, and weather conditions have temporal patterns. Sunrise and sunset create daily patterns, and yearly patterns are correlated with the regional climate. Prediction targets and a few meteorological variables related to the targets (e.g., wind speed and direction) are insufficient in providing contextual information on the weather in a region. Thus, analyzing spatiotemporal correlations between various meteorological variables with an end-to-end network will improve the performance of weather forecasting models. In addition, as discussed above, models that are not based on atmospheric knowledge have limited model interpretability. Although several existing studies have attempted to combine multiple features, they did not closely examine the effects of combining the three features on weather forecasting with a case study of solar irradiance.

Therefore, we first developed a novel solar irradiance forecasting model that considers (i) temporal patterns of meteorological variables, (ii) spatial influences between observation stations, and (iii) correlations among a variety of meteorological variables. The weather data were represented as a graph, with the observation stations as nodes, the spatial adjacency of the stations as edges, and meteorological variables as attributes. Thus, the graph exhibits static structures and dynamic attributes. Then, we extended the attribute-augmented spatiotemporal GCN (AST-GCN) model [51], which considers both static and dynamic attributes, to analyze spatiotemporal correlations between multiple meteorological variables. The AST-GCN model consists of graph convolution layers and recurrent layers, which extract the spatial and temporal features of the dynamic networks, respectively. However, the graph convolution layers also analyze the temporal changes in the dynamic attributes using a fixed-length window. We call the proposed model for multivariate spatiotemporal analysis of meteorological data “Multi-attributed Spatio-Temporal GCN (MST-GCN).”

To examine the effects of the feature combination, we compared the performance of the proposed model with baseline models, which are based on each part of the three features, by adjusting the prediction sequence lengths, seasons, weather conditions, etc. Based on this comparison, we attempted to validate the following research questions:

- RQ1. Weather conditions of spatially adjacent observation stations influence each other, and the influence is significant in predicting solar irradiance.
- RQ2. Temporal changes in historical weather data are effective in solar irradiance forecasting.
- RQ3. Meteorological variables observed at a station have correlations with future solar irradiance of the station.

The performance of the proposed and existing models demonstrated the contribution of each feature to the aspects of weather forecasting. The performance comparison between the models showed that the spatial, temporal, and multivariate features complemented each other and were synergistic. The main contributions of this study can be summarized as follows:

- We propose MST-GCN, which allows for spatiotemporal analysis of dynamic multi-attributed networks to conduct day-ahead hourly solar irradiance forecasting for multiple stations. Our proposed model consists of GCN layers for spatial features, GRU layers for temporal features, and multi-attribute fusion modules for multivariate features to fuse the three features of meteorological data.
- We demonstrated the superiority of MST-GCN in terms of forecasting performance and stability over the baseline models, including T-GCN (spatiotemporal), GRU (temporal), GCN (spatial), and MLP (multivariate) with intensive experiments. Furthermore, we verified the above research questions, RQ1, RQ2, and RQ3, by comparing T-GCN with GRU, T-GCN with GCN, and MST-GCN with T-GCN, respectively.

The remainder of this paper is organized as follows: Section 2 describes the acquisition and pre-processing of the meteorological data used in this study. Section 3 presents the proposed solar irradiance forecasting model. Section 4 details the experimental procedures and results used to evaluate the performance and practicality of the proposed model. Section 5 presents the concluding remarks and discusses the limitations and future directions of this study.

## 2. Data Acquisition and Preprocessing

This section describes the procedures for acquiring meteorological data used to evaluate the proposed model and validate the research questions.

### 2.1. Resource Data

There are two methods for measuring solar irradiance. The first method uses a pyrometer, and the other indirectly estimates solar irradiance by analyzing satellite images. Although the pyrometer can accurately measure the amount of insolation per hour, it has disadvantages in terms of the high cost of the measurement system and the limited measurable range [30,52,53]. Satellite image analysis has advantages in observing solar irradiance over a wide area. However, this method also has difficulties in real-time estimation, owing to the characteristics of satellite imaging [54,55]. In addition, because satellite images are taken over clouds, cloudiness can cause images to be much less accurate than images from ground observations. Owing to this inherent limitation, satellite image analysis showed a relatively lower accuracy than the pyrometer. Uncertainties in the observation data also affect the performance of solar irradiance forecasting models using measurements. Most of the existing forecasting models based on satellite images have lower and less stable accuracies than pyrometer-based models [56,57]. Thus, we acquired meteorological data collected by automated surface observing systems (ASOS), which are based on a pyrometer and more accurate than satellite image analysis, to reduce uncertainties caused by input variables.

**Figure 1:**
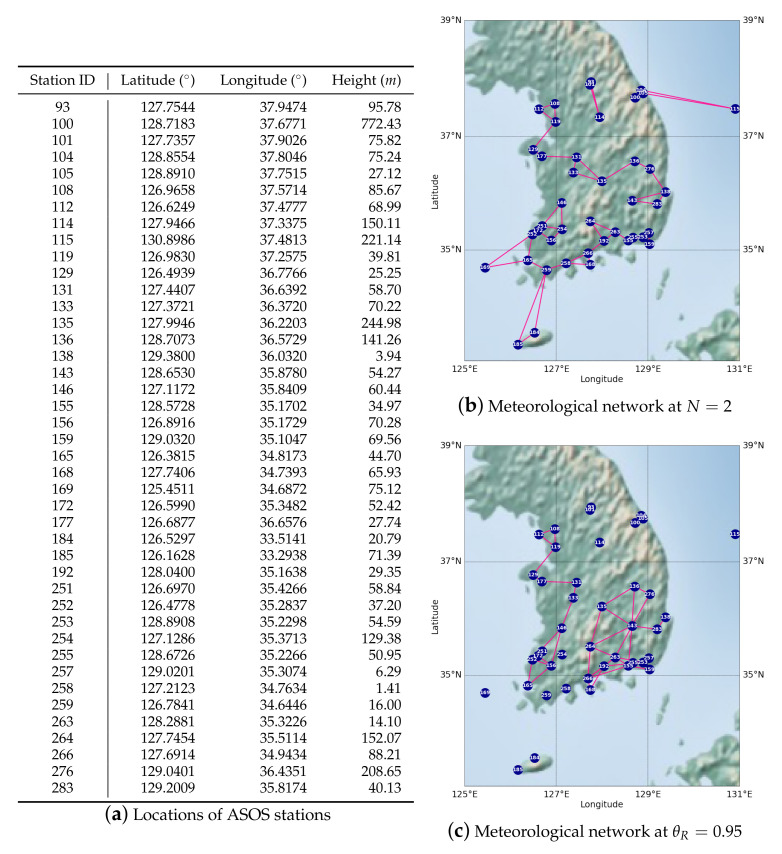
Information of automated surface observing systems (ASOS) stations and examples of meteorological networks. (**a**) presents geographic coordinates of ASOS stations. (**b**,**c**) show meteorological networks composed based on geographical distances between the ASOS stations and based on correlations between historical solar irradiance of the stations, respectively. *N* indicates the number of neighboring stations, and $\theta_{R}$ denotes the threshold of correlations between solar irradiance histories.

The ASOS Programme is a joint effort of the National Weather Service (NWS), Federal Aviation Administration (FAA), and Department of Defense (DOD). The ASOS serves as the nation’s primary weather-observing surface network. This system was designed to support weather forecasting and aviation operations. Simultaneously, the ASOS supports the needs of meteorological, hydrological, and climatological research communities [58,59]. The ASOS conducts time-synchronized ground observations at every participating observatory for obtaining time-sequential data for atmospheric conditions. In addition, the system automatically measured meteorological variables using synoptic meteorological observation equipment. The observational data were accessible through a public repository (https://data.kma.go.kr/cmmn/main.do (accessed on 15 August 2022)). Among the ASOS observation stations, we selected 42 stations measuring solar irradiance since 2017 and located in the Korean Peninsula, as shown in Figure 1.

### 2.2. Meteorological Variables

Solar irradiance ($S_{r}$) is closely related to the geographical factors of observatories, the date and time of observations, and other meteorological variables (e.g., cloudiness and precipitation). From a geographical perspective, solar irradiance varies with latitude and longitude. As discussed in Section 1, the spatial adjacency between observatories indicates that weather contexts can influence each other. In addition, time-sequential analysis can establish daily and seasonal patterns of solar irradiance. Therefore, utilizing these spatial and temporal features can improve the performance of solar irradiance forecasting models [60,61]. From an atmospheric standpoint, weather contextual information, which can be inferred from meteorological variables, is significant for predicting solar irradiance [30]. For example, if it is a cloudy day, the sun is blocked and the amount of insolation reaching the ground decreases. Therefore, to consider weather contexts with high variability, we gathered meteorological parameters correlated with solar irradiance, and spatiotemporal parameters. Among the variables in the ASOS data, we selected 17 variables in the three categories as input parameters for the proposed model, as listed in Table 1.

### 2.3. Data Preprocessing

The ASOS data have a significant number of missing values, and interpolating the omitted observations can cause uncertainties and affect the performance of the forecasting models. Thus, for fair evaluation and validation, we removed the variables and adjusted the observation period for avoiding missing values. However, a few values are significantly correlated with solar irradiance and are not difficult to reliably substitute for omitted values. For precipitation, we checked records from the Korea Meteorological Administration for regions where observation stations with missing precipitation values were located. If there was no precipitation when missing values occurred, we replaced them with zero. We examined sunrise and sunset times in cases of missing sunshine duration and solar irradiance. Because insolation cannot exist between sunset and sunrise (e.g., 21:00 KST to 05:00 KST), we replaced the missing sunshine duration and solar irradiance values in the period with zero. As a result, we gathered hourly observation data for four years (from 1 January 2017 to 31 December 2020), including the 17 meteorological variables observed at the 42 observatories. The first three years of data were used to train the proposed and baseline models, and the remaining year was used for model evaluation.

## 3. Methods

We propose a novel solar irradiation forecasting model that considers (i) spatial features, (ii) temporal features, and (iii) correlations between meteorological variables. First, we represented the ASOS data as undirected networks with multiple dynamic attributes. We then modified and extended the existing spatiotemporal GCN models [51,62] to analyze the spatial and temporal correlations between these dynamic attributes.

### 3.1. Meteorological Networks

The proposed model conducts solar irradiance forecasting by analyzing (i) spatial correlations between ASOS stations, (ii) historical patterns of meteorological variables, and (iii) correlations of solar irradiance with the variables. These three viewpoints will enable the proposed model to establish weather contexts at each ASOS station and to predict future weather by understanding the spatiotemporal influences between the stations. First, we represented the spatial correlations as an undirected network and historical meteorological variables observed at each ASOS station as the dynamic node attributes of the network.

Most of the existing studies defined correlations between meteorological observation sites by using mutual information [49,63] and the PCC [43,45]. However, the influence between the two observation sites will have inconsistent time lags according to distances, landforms, weather contexts, and so on. Moreover, mutual information and correlation coefficients are not proper metrics for detecting the influence of dynamic time lags. Therefore, we defined the correlations between ASOS stations using geographical distances. Then, the relative correlations among the adjacent stations can be learned by the GCN layers in the proposed model. For the same reason, although existing studies [43,45,49,63] defined the adjacency between stations by using minimum thresholds for correlations, we searched for *N*-nearest neighborhoods of the stations according to the distances. We call the network representing dynamic weather data ‘meteorological network’, and it can be defined as follows:

**Definition** **1** (Meteorological Network). *The geographical adjacency of the ASOS stations is described as N=(V,E), where $V=\{v_{1},\cdots ,v_{V}\}$ is the set of stations and V is the number of stations. $E∋e_{i,j}$ is the set of edges, where $v_{j}$ is one of the N-nearest neighbors of $v_{i}$. In addition, each node had meteorological variables (listed in Table 1) collected by the corresponding ASOS stations as dynamic attributes. Thus, the structure of the static meteorological network can be represented as an adjacency matrix $A\in R^{V\times V}$. Then, the dynamic attributes can be represented as a sequence of matrices, $X=\langle X_{1},\cdots ,X_{T}\rangle$, where T denotes the number of time points and $X_{t}\in R^{K\times V}$ refers to node attributes at time t when K is the number of meteorological variables.*

**Figure A1:**
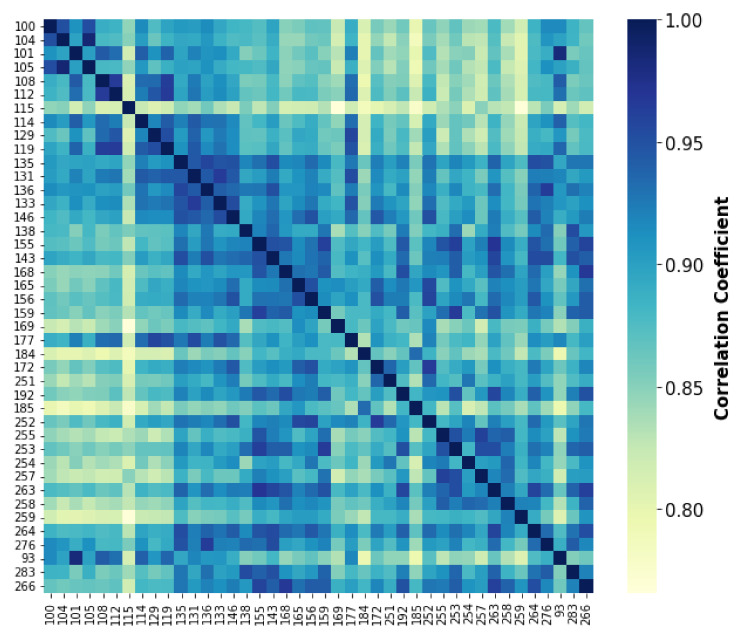
A heat map for Pearson correlation coefficients between historical solar irradiance (January 2017–December 2019) of the ASOS stations. The darker colors indicate that solar irradiance of two ASOS stations were more correlated with each other.

Figure 1b presents an example of a meteorological network when the number of neighborhoods (*N*) is two. The proposed approach assigns at least *N* candidate stations that can be correlated with the target station, assuming that we do not know the degree of correlation at this moment. Figure 1c shows the case with the minimum threshold ($\theta_{R}$) for the PCC (Figure A1). This approach allows stations to have a flexible-size neighborhood, but there can be isolated stations; models cannot learn the spatial correlations of those stations. Similarly, when *N* is too large or $\theta_{R}$ is too small, we can miss the spatial correlations between stations. In the opposite case, the model is confused by overabundant information. In Section 4.4.2, we discuss the advantages and disadvantages of the two approaches by evaluating the proposed model based on *N* and $\theta_{R}$.

The node attributes at time point *t* ($X_{t}$) consist of solar irradiance, which is the forecasting target, and other meteorological variables related to solar irradiance. The solar irradiance is defined as follows:

**Definition** **2** (Solar Irradiance). *Solar irradiance at a station at time t is viewed as one of the node attributes of a meteorological network. Thus, $X_{t,S_{r}}\in R^{V}$ is a row vector of $X_{t}$ that represents the solar irradiance of all stations at time t. In addition, the ith component of $X_{t,S_{r}}$ ($S_{r}\left(t,i\right)$) corresponds to the solar irradiance degree at the ith station at time t.*

The proposed model predicts future solar irradiance by analyzing previous solar irradiance and meteorological variables. The spatiotemporal correlations of meteorological variables with solar irradiance will enable the proposed model to understand weather contexts that can affect solar irradiance. Although we acquired the 16 variables listed in Table 1 from the ASOS data, the last column of the table says that not all the variables have explicit correlations with solar irradiance. Similar to the adjacency of observation stations, the PCC will be insufficient for establishing the spatiotemporal correlations of solar irradiance with meteorological variables. However, omitting highly correlated variables can hinder the proposed model from recognizing weather contexts, and appending extraneous variables can confuse the model. Thus, the meteorological variables can be defined based on the threshold for their PCC with solar irradiance ($\theta_{V}$) as follows:

**Definition** **3** (Meteorological Variables). The remaining node attributes are multiple variables that correlate with solar irradiance and reflect the weather context. When $K=\{k_{1},\cdots ,k_{K}\}$ is the set of all available meteorological variables and r(·,·) indicates the PCC between two variables, the node attributes can be formulated as $K^{*}=\{k_{j}\left|k_{j}\in K,r\left(k_{j},S_{r}\right)\geq \theta_{V}\right\}$. In addition, similar to solar irradiance ($S_{r}$), when $k_{j}\left(t,i\right)$ refers to the value of $k_{j}$ at the ith station at time t, $X_{t,k_{j}}$ indicates a vector representing the values of $k_{j}$ at time t at every observation station. By concatenating $X_{t,k_{j}},\forall k_{j}\in K^{*}$, we can compose an attribute matrix at time t, $X_{t}$.

In Section 4.4.1, we evaluate the proposed method to compose a set of meteorological variables by adjusting $\theta_{V}$. Therefore, solar irradiance forecasting, which models temporal and spatial dependencies between solar irradiance and meteorological variables, can be defined as learning a mapping function *f* based on the meteorological network N=〈A,X〉 that consists of the static adjacency matrix A and the dynamic node attributes X. When $L_{p}$ and $L_{o}$ are the prediction and observation sequence lengths, respectively, the forecasting procedure can be formulated as:

(1) $$\begin{array}{c} \langle X_{t+1,S_{r}},\cdots ,X_{t+L_{p},S_{r}}\rangle =f\left(A,\langle X_{t-L_{o}+1},\cdots ,X_{t}\rangle \right). \end{array}$$

### 3.2. Multi-Attributed Spatio-Temporal Graph Convolutional Network

**Figure 2:**
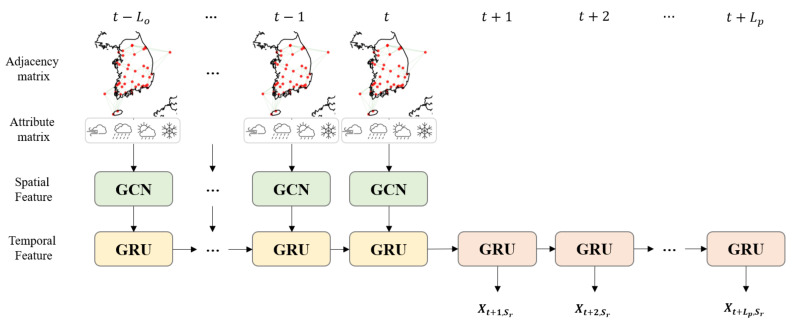
An overview of the proposed model. Spectral graph convolution layers extract structural features of meteorological networks and spatial correlations of meteorological variables with adjacent ASOS stations at each time point. GRU layers learn temporal correlations of solar irradiance with other meteorological variables by analyzing feature vectors that come from the graph convolution layers.

The proposed model aims to discover the spatio-temporal correlations of solar irradiance with multiple meteorological variables. The existing spatio-temporal GCN models [51,62,64,65,66] have barely paid attention to dynamic changes in node attributes. Thus, we propose a novel spatio-temporal GCN model that can consider multiple dynamic node attributes by extending the AST-GCN [51], which considers deals with both static and dynamic attributes. The proposed model mainly consists of GCN layers and GRU layers. The GCN layers focused on extracting spatial features from snapshots of the meteorological network at each time point. Then, the GRU layers then analyze temporal changes in the spatial features for predicting to predict future solar irradiance. We call the proposed model ‘MST-GCN (Multi-attributed Spatio-Temporal Graph Convolutional Network)’, and the structure of the model are illustrated in Figure 2.

#### 3.2.1. Multi-Attribute Fusion

This study represents multiple meteorological variables observed at each station as attributes of corresponding nodes to infer micro- and macro-weather conditions and their spatiotemporal correlations. Thus, the adjacency matrix A is static and represents only the geographical adjacency of the stations. The node attributes $X=\langle X_{1},\cdots ,X_{T}\rangle$ are dynamic and expressed as a sequence of attribute matrices at each time point. The simplest approach for feeding N=〈A,X〉 into spatiotemporal GCN models is using $N_{t}=\langle A,X_{t}\rangle$ as inputs to GCN layers and learn temporal changes in feature vectors for $N_{t-L_{o}}$ to $N_{t}$ on the GRU layers. However, this approach overlooks the spatial influences of weather conditions on observation stations, which are not immediate. To analyze the spatial influences with agnostic and unfixed time lags, we let the GCN layers observe multiple time points using a fixed-length sliding window. In addition, the GRU layers compress the input features at multiple time points into fixed-length vectors, which causes information loss. This approach can reduce the risk of information loss by sharing the burden of temporal analysis with the GCN layers. When the length of the sliding window is *l*, the input network of the GCN layer at time *t* can be formulated as:

(2) $$\begin{array}{c} N_{t}=\langle A,X_{t-l+1}^{t}\rangle =\langle A,\langle X_{t-l+1},\cdots ,X_{t}\rangle \rangle , \end{array}$$

where $X_{t-l+1}^{t}\in R^{lK\times V}$ indicates the concatenation of attribute matrices within the window. Although Zhu et al. [51] suggested this approach, they did not evaluate its effectiveness in analyzing multiple dynamic attributes. Section 4.2 focuses on verifying the effectiveness of enabling the GCN layers to conduct a spatiotemporal analysis by comparing it with T-GCN [62]. We heuristically set the window size *l* as six. The proposed model aims for day-ahead hourly forecasting, and the 42 ASOS stations used in this study are densely located in the southern part of the Korean Peninsula. Thus, we assumed that l=6 is a sufficient time period for establishing the inter-station spatial influences required for predicting the weather tomorrow.

#### 3.2.2. Spatial Dependency Modeling

Discovering the spatial influences between the weather contexts of observation stations is significant for predicting future weather contexts and forecasting solar irradiance. Graph convolutional network (GCN) models, which are the generalization of convolutional neural network (CNN) models to graph-structured data, have been shown to be effective for analyzing the propagation of node features between adjacent nodes. The proposed model employs the spectral graph convolution method proposed by Kipf and Welling [67], which improves the computational complexity of the existing spectral GCN models. This method updates node features to smooth (and denoise) the features of neighboring nodes by conducting convolution operations in the spectral domains. Thus, convolution filters extract spatial features between nodes by analyzing them and their first-order neighborhoods. By stacking multiple GCN layers, we can obtain the representation of each node while considering the influence of adjacent nodes. This study initially sets the node features as meteorological variables, including solar irradiance, from t−l+1 to *t*. Then, the GCN layers generate representations of weather contexts at each station at time *t* based on spatial influences between the weather contexts of adjacent stations during the time period [t−l+1,t]. The GCN layer in the proposed model can be formulated as:

(3) $$\begin{array}{ccc} & & H_{t}^{\left(n\right)}=\sigma \left(\hat{A}H_{t}^{\left(n-1\right)}\theta^{\left(n\right)}\right),\;H_{t}^{\left(0\right)}=X_{t-l+1}^{t},\\ & & \hat{A}={\tilde{D}}^{-\frac{1}{2}}\tilde{A}{\tilde{D}}^{-\frac{1}{2}}, \end{array}$$

where $\tilde{A}=A+I$ denotes an adjacent matrix with self-connection structures. $I\in R^{V\times V}$ is an identity matrix $\tilde{D}$ denotes the degree matrix of $\tilde{A}$ (${\tilde{D}}_{i,i}=\sum_{\forall j}{\tilde{A}}_{i,j}$). $H_{t}^{\left(n\right)}$ is the feature matrix generated by the *n*th GCN layer at time *t*. $H_{t}^{\left(0\right)}$ represents the initial node feature at time *t*. $\theta^{\left(n\right)}$ refers to the convolution filter of the *n*th layer. Finally, σ(·) denotes the activation function used for nonlinear modeling. Therefore, each GCN layer linearly transforms the feature matrix ($H_{t}^{\left(n-1\right)}$) using $\theta^{\left(n\right)}$ where each column of $H_{t}^{\left(n-1\right)}$ represents the weather context of a station, and aggregates these features to compose the feature matrix for the next layer ($H_{t}^{\left(n\right)}$) according to $\hat{A}$. Although we provide the meteorological variables during the time windows ($H_{t}^{\left(0\right)}=X_{t-l+1}^{t}$) to the GCN layers, it cannot allow the model to determine the temporal order of the variables. However, the model can learn the spatial correlations among variables at each time point in the windows. The GRU layers then discover more distinctive spatiotemporal correlations from temporal changes in the sequence of feature matrices ($\langle H_{1}^{\left(n\right)},\cdots ,H_{T}^{\left(n\right)}\rangle$).

As discussed in the previous section, the meteorological network had 42 nodes (stations), and the out-degrees of the nodes were at least *N*. The edge density of the meteorological network is far higher than that of conventional networked data such as bibliographic networks. Because aggregating information of a few hops will reach across the network (or its sub-networks), simply stacking a few GCN layers can cause an over-smoothing problem. Thus, we heuristically set the number of GCN layers to 2. In addition, we used a rectified linear unit (ReLU) as the activation function.

#### 3.2.3. Temporal Dependency Modeling

The node representations extracted by the GCN layers reflect the spatiotemporal correlations between the meteorological variables. However, extending the window size (*l*) as long as the observation period ($L_{o}$) provides overabundant information that exceeds the learning capabilities of the GCN layers. Thus, time-varying representations with short-term spatiotemporal features are fed into GRU layers to establish temporal dependencies between meteorological contexts in the long term. The GRU layers can be regarded as the compositions of the reset and update gates. When $r_{t}$ and $u_{t}$ are the reset gate and update gate at time *t*, respectively, $r_{t}$ is used to control the amount of information of the previous time points (i.e., the output at the previous time point, $h_{t-1}$) that will be remembered or forgotten. Likewise, $u_{t}$ controls how much past information should be reserved. $r_{t}$ combines the input feature vector on *t* with $h_{t-1}$ to compose the current cell state, $c_{t}$. The final output on *t* ($h_{t}$) is then derived by combining $c_{t}$ and $h_{t-1}$ using $u_{t}$. This procedure can be formulated as follows:

(4) $$\begin{array}{ccc} u_{t} & = & \sigma (\theta_{u}\left[H_{t}^{\left(N\right)},h_{t-1}\right]), \end{array}$$

(5) $$\begin{array}{ccc} r_{t} & = & \sigma (\theta_{r}\left[H_{t}^{\left(N\right)},h_{t-1}\right]), \end{array}$$

(6) $$\begin{array}{ccc} c_{t} & = & \phi (\theta_{c}\left[H_{t}^{\left(N\right)},\left(r_{t}*h_{t-1}\right)\right]), \end{array}$$

(7) $$\begin{array}{ccc} h_{t} & = & u_{t}*h_{t-1}+\left(1-u_{t}\right)*c_{t}, \end{array}$$

where σ(·) denotes a sigmoid function. ϕ(·) denotes the hyperbolic tangent function. * refers to the Hadamard product. $H_{t}^{\left(N\right)}$ is a spatial feature matrix extracted from $N_{t}$ using the last GCN layer. $\theta_{u}$, $\theta_{r}$, and $\theta_{c}$ are learnable weight matrices. We stacked two GRU layers above the GCN layers, and one fully connected layer was used to predict the future solar irradiance from $h_{t}$ with linear activation.

The goal of solar irradiance forecasting is to make the prediction result approximate the actual weather conditions as closely as possible. Thus, the objective of the proposed model was to minimize the prediction error. The error was measured by the L2 loss, and the objective function can be formulated as:

(8) $$\begin{array}{c} L=\sum_{\tau \in [t+1,t+L_{p}]}{∥X_{\tau ,S_{r}}-{\hat{X}}_{\tau ,S_{r}}∥}_{2}^{2}+\lambda {∥\theta ∥}_{2}^{2}, \end{array}$$

where $X_{\tau ,S_{r}}$ and ${\hat{X}}_{\tau ,S_{r}}$ are the actual and predicted solar irradiances at time τ, respectively, and λ is a hyperparameter that controls the regularization rate.

## 4. Evaluation

This section presents the experimental procedures and results for evaluating the prediction performance of the proposed model and validating the research questions underlying the proposed approaches. First, we compared the performance of the proposed model with baseline models, including both conventional regression models (e.g., HA, ARIMA, VAR, and SVR) and neural network models (e.g., MLP, GCN, GRU, and T-GCN). We also examined the performance of the proposed and existing models in terms of long-term predictions. This experiment demonstrates the practicality of the proposed model and shows whether the models understand the dynamic changes in weather contexts. Subsequently, we examined the stability of the forecasting models by comparing their performance variations according to cloudiness and months. Finally, the proposed model has several hyperparameters that determine the meteorological variables and neighboring stations that were used for forecasting. We evaluated the sensitivity of the proposed model by assessing its performance according to the hyperparameters.

### 4.1. Experimental Settings

This section describes the experimental settings, including the datasets, accuracy metrics, hyperparameter settings, and the comparison groups. We acquired meteorological observation data from 42 ASOS stations for four years (1 January 2017 to 31 December 2020), as described in Section 2.1. We collected hourly solar irradiance data during the observation period and the 16 meteorological variables correlated with solar irradiance, as listed in Table 1. The observation data of the first three years (1 January 2017 to 31 December 2019) were used as the training dataset. We then evaluated the proposed model using the remaining years (1 January 2020 to 31 December 2020). For the experiments, every meteorological variable was normalized to [0,1]. We predicted the solar irradiance of the next $L_{p}\in \left\{1,2,3,4,5,6,12,24\right\}$ h at each ASOS station by analyzing the solar irradiance and meteorological variables during the previous $L_{o}\in \left\{12,24\right\}$ h.

In this study, we used six accuracy metrics to evaluate the performance of solar irradiance forecasting models: root mean square error ($E_{2}$), mean absolute error ($E_{1}$), normalized mean square error ($NE_{2}$), accuracy (*A*), R-squared ($R^{2}$), and variance (σ). These metrics can be measured as follows:

(9) $$\begin{array}{ccc} & & E_{2}={\left[\frac{1}{T}\sum_{\forall t}{\left(Y_{t}-\hat{Y_{t}}\right)}^{2}\right]}^{\frac{1}{2}},\;E_{1}=\frac{1}{T}\sum_{\forall t}\left|Y_{t}-\hat{Y_{t}}\right|,\;NE_{2}=\frac{E_{2}}{\bar{Y}},\\ & & A=1-\frac{{∥Y-\hat{Y}∥}_{F}}{{∥Y∥}_{F}}=1-\frac{E_{2}}{{∥Y∥}_{F}},\;R^{2}=1-\frac{\sum_{\forall t}{\left(Y_{t}-\hat{Y_{t}}\right)}^{2}}{\sum_{\forall t}{\left(Y_{t}-\bar{Y}\right)}^{2}},\;\sigma =1-\frac{\sigma \left(Y-\hat{Y}\right)}{\sigma \left(Y\right)} \end{array}$$

where $Y_{t}$ and $\hat{Y_{t}}$ indicate the observed and predicted solar irradiances at time *t*, respectively. *T* denotes the total number of time-points. ${∥\cdot ∥}_{F}$ refers to the Frobenius norm; *Y* denotes the average $Y_{t}$ for ∀t∈[0,T]; and σ(·) indicates variance. $E_{2}$ and $E_{1}$ show the average errors of the forecasting models, and a comparison of $E_{2}$ and $E_{1}$ presents the variation in the errors. $NE_{2}$, *A*, and $R^{2}$ are normalized errors. $NE_{2}$ normalizes $E_{2}$ based on average observed solar irradiance. *A* considers the deviation of both the observed and predicted values by comparing $E_{2}$ with the root square mean of the observed values. $R^{2}$ normalizes the mean squared error based on the variance of the observation. Finally, σ compares the variance of the errors with that of the observation.

We compared the performance of the proposed model with that of the following baseline models:

- HA (history average) [68] uses the average solar irradiance in the historical periods as the prediction.
- ARIMA (autoregressive integrated moving average) [27] fits the observed solar irradiance into a parametric model to predict future solar irradiance.
- VAR (vector autoregression) [69] fits the observed solar irradiance and other multiple meteorological variables into a parametric model to predict future solar irradiance.
- SVR (support vector regression) [70] searches an optimal linear function of meteorological variables for solar irradiance with hinge loss. We use the linear kernel and the penalty term is 0.001.
- MLP (multi-layer perceptron) [71] is a typical neural network model, which consists of five fully-connected layers. The hidden layers included 256, 128, 64, 32, and 16 nodes, respectively. We used ReLu activation function and RMSProp optimizer.
- GCN [67] is a graph neural network, which extracts spatial features from networked data by transforming and aggregating feature vectors of nodes and their neighborhoods. We applied a two-layered GCN model to predict $\langle X_{t+1,S_{r}},\cdots ,X_{t+L_{p},S_{r}}\rangle$ by analyzing A and $\langle X_{t-L_{o}+1,S_{r}},\cdots ,X_{t,S_{r}}\rangle$ without considering the temporal order.
- GRU [72] is a RNN model that improves the long-term dependency problem of the conventional RNN and reduces parameters of LSTM. We used a three-layered GRU model with 64 hidden units to predict $\langle S_{r}\left(t+1,i\right),\cdots ,S_{r}\left(t+L_{p},i\right)\rangle$ at each station $v_{i}$ by analyzing only temporal changes in $\langle S_{r}\left(t-L_{o}+1,i\right),\cdots ,S_{r}\left(t,i\right)\rangle$.
- T-GCN [62] is a spatio-temporal graph neural network, which is a combination of GCN and GRU, in order to analyze networks with static structures and dynamic attributes. This model uses a GCN layer to extract spatial features ($H_{\tau }^{\left(N\right)}$) from A and $X_{\tau ,S_{r}}$ on each time point $\tau \in [t-L_{o}+1,t]$ and a GRU layer to predict $\langle X_{t+1,S_{r}},\cdots ,X_{t+L_{p},S_{r}}\rangle$ by analyzing temporal changes from $H_{t-L_{o}+1}^{\left(N\right)}$ to $H_{t}^{\left(N\right)}$.

The proposed model was implemented using TensorFlow in Python. We used a hyperbolic tangent function as the activation function of the output layer, ReLu function for the hidden layers, and Adam optimizer [73]. We conducted a grid search for the proposed model’s hyperparameters: number of epochs: 500 to 3000 with a step size of +500, learning rate: 0.0001 to 0.01 with a step size of ×10, batch size of 32 to 512 with a step size of ×2, number of GRU hidden units: 8 to 128 with a step size of ×2, and number of neighboring observation sites: 1 to 9 with a step size of +1. The proposed model had the best accuracy for the number of epochs =1000, learning rate =0.001, batch size =128, number of GRU hidden units =64, and number of neighboring observation sites =2. The implementation of the proposed model is available in the GitHub repository (https://github.com/higd963/MST-GCN (accessed on 15 August 2022)).

### 4.2. Effectiveness of the Proposed Model

This section evaluates the proposed model by comparing its accuracy with that of the baseline models. The models were trained to predict the solar irradiance at time *t* by analyzing the previous solar irradiance from t−1 to t−12. We also compared the accuracy of the models on multivariate analysis with univariate analysis to demonstrate that the proposed model is more effective for analyzing correlations between multiple meteorological variables than existing models. Additionally, HA and ARIMA cannot deal with multivariate features, and the proposed model and VAR are not designed for univariate analysis. Thus, we could not assess model accuracy for those cases and remained as blanks on Table 2, which lists the experimental results.

The proposed method outperformed the existing models in every evaluation metric. In addition, the existing models exhibited a significant performance decrement in the multivariate analysis compared to the univariate analysis. This result is unexpected because T-GCN [62] and the proposed model do not have significant differences in their model structures. When we modified T-GCN to consider multiple meteorological variables, the major difference between the two models is that the proposed model observes samples from time t−m to *t* with an m+1-length window on time *t*, but T-GCN only considers samples at *t*. Thus, the graph convolution layers in the proposed model can extract spatiotemporal features from meteorological data. Otherwise, the graph convolution layers and recurrent layers in the T-GCN focus only on spatial and temporal features, respectively. Thus, we can assume that spatiotemporal analysis is more effective for discovering correlations between meteorological variables than aggregating spatial features using recurrent layers (RQ1 and RQ2).

The neural network models with temporal features (e.g., T-GCN and GRU) outperformed the other models in univariate analysis. However, in the multivariate case, GRU exhibited a worse performance than GCN. Although the recurrent layers could be effective for discovering daily patterns of sunshine, stacking the recurrent layers was not sufficient to establish and utilize the correlations between meteorological variables. This point was also shown in that T-GCN underperformed GRU in the univariate case, which was the opposite in the multivariate case. All existing models exhibited significantly worse performance on multivariate analysis than on univariate analysis. This result might be caused by limitations in the learning capabilities of the models, the same as with the GRU.

The deep learning-empowered models significantly outperformed the conventional regression models in both the univariate and multivariate cases, excluding SVR. SVR had the best performance in univariate analysis considering $E_{2}$ and $NE_{2}$. On the other metrics, SVR showed the second-best performance, with a slight difference. This is an interesting result in that SVR can comprehend temporal changes in solar irradiance as much as GRU and T-GCN. However, it exhibited a catastrophic performance decrease in the multivariate case. Because the models conducted one-hour-ahead prediction by analyzing previous solar irradiance during 12 h, SVR could be sufficient for applying daily patterns to the meteorological contexts of each day. Meteorological variables have complicated spatiotemporal correlations, requiring forecasting models with highly expressive capabilities, which conventional models cannot support.

As there have been studies on hourly day-ahead forecasting of solar irradiance [74], we examined the practicality of the proposed model by comparing the accuracy of the proposed and existing models according to various prediction and observation periods. Using two observation periods, $L_{o}=$ 12 and 24, we adjusted the prediction periods ($L_{p}$) in [1,6] and {1,3,6,12,24}, respectively. In the above experiment, the conventional regression models significantly underperformed the deep learning-empowered models, and none of the existing models properly conducted multivariate analysis. Thus, the following experiments employed only deep neural networks (e.g., MLP, GCN, GRU, and T-GCN) trained for univariate analysis as baseline methods. Table 3 and Figure A3 show the results of this experiment.

The proposed model exhibited the highest accuracy for most cases and metrics. The performance improvement was more noticeable in the long-term prediction than in the short-term prediction because the proposed model showed consistently high accuracy according to $L_{p}$ in both $L_{o}=12$ and =24 cases. However, in the short-term prediction, GRU performed similarly to the proposed model, even higher for $L_{o}=24$ and $L_{p}=\left\{1,3\right\}$. As discussed in the previous experiment, the GRU can be effective for discovering periodic patterns of solar irradiance. In short-term prediction (far shorter than a day), observing the discovered patterns would be more significant than recognizing weather contexts by understanding spatiotemporal correlations between the meteorological variables (RQ2). This point would be the opposite in long-term prediction.

Similarly, in both $L_{o}=12$ and =24 cases, T-GCN performed better than GRU in long-term prediction and worse than GRU in short-term prediction. In the $L_{o}=12$ case, reversal occurred at $L_{p}=3$. However, in the $L_{o}=24$ case, the reversal occurred at $L_{p}=24$, which is much later than in the $L_{o}=12$ case. There are two reasons for this result. First, the GRU can learn daily solar irradiance patterns more deeply at $L_{o}=24$ than at $L_{o}=12$. In addition, the T-GCN would not be able to recognize weather context information as accurately as the proposed model because it is a univariate model and cannot analyze correlations between the various meteorological variables. Furthermore, a comparison of the GCN with GRU showed a similar result to the above comparisons only in the $L_{o}=12$ case from $L_{p}=4$. However, GRU had a higher σ than GCN in most cases, excluding when $L_{o}=12$ and $L_{p}=5$. In addition, the GRU significantly outperformed the GCN in all cases and metrics in the $L_{o}=24$ case. The GCN and GRU focus only on spatial correlations and temporal changes in solar irradiance, respectively. The results of the three comparisons indicate that weather contexts discovered from the spatial features are effective for long-term prediction (RQ1). Simultaneously, the spatial features could not show their effectiveness without combining with the temporal features (RQ2), and the combination of spatiotemporal analysis and multivariate analysis could enhance model capabilities for understanding weather contexts (RQ3). Additionally, the performance decrement of the GRU would not come from the long-term dependency problem because the proposed model and T-GCN also learn temporal changes in spatial features extracted by GCN layers using GRU layers.

MLP significantly underperformed the other models. Although MLP exhibited consistent performance according to changes in $L_{p}$, its prediction results are difficult to be used for forecasting systems, considering that the observed solar irradiance values were normalized into [0,1]. Although MLP analyzes correlations between the meteorological variables, the multivariate analysis without spatial and temporal features could not effectively recognize weather contexts.

From these experimental results, we can discover that (i) spatial correlations between observation sites are essential for consistent forecasting performance on both long-term and short-term prediction (RQ1), (ii) in short-term prediction, periodic patterns are more effective than the other features (RQ2), (iii) spatial correlations show their worth when used with the periodic patterns (RQ1 and RQ2), and (iv) correlations between multivariate variables could not show high accuracy solely but exhibited its effectiveness when used with the others (RQ3).

### 4.3. Stability of the Proposed Model

This section presents the performance stability of the proposed model by comparing its accuracy fluctuation according to weather conditions with those of the baseline models (e.g., GCN, GRU, and T-GCN). Solar irradiance is affected by various weather factors, such as cloudiness, and seasons are correlated with the annual patterns of solar irradiance and weather. Therefore, we first examined the forecasting models’ performance at every cloudiness level as a representative factor affecting the solar irradiance. The monthly performance of the models was then evaluated for determining the seasonal influence on solar irradiance and the forecasting models.

#### 4.3.1. Performance Variation according to Cloudiness

Solar irradiance showed relatively consistent patterns on clear days, and sunny days were more frequent than cloudy days. Therefore, we must evaluate whether the proposed model can achieve high accuracy regardless of cloudiness for examining the practicality of the model. We classified cloudiness into 10 degrees, and our data samples were segmented according to the degree of cloudiness. Subsequently, we evaluated the performance of the proposed and existing deep-learning-empowered models within each segment of the dataset. Table 4 and Figure A4 list the experimental results.

**Figure A2:**
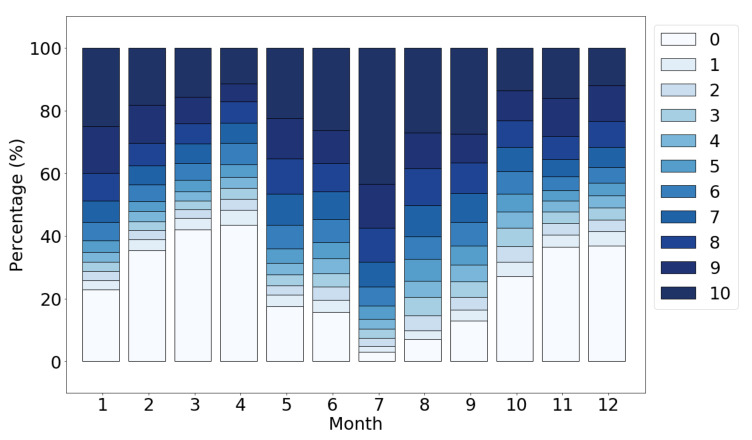
Monthly distributions of cloud cover in our experimental dataset (January 2017–December 2020). Cloud cover had imbalance distributions within each month. Moreover, the distributions were different for each month.

**Figure A3:**
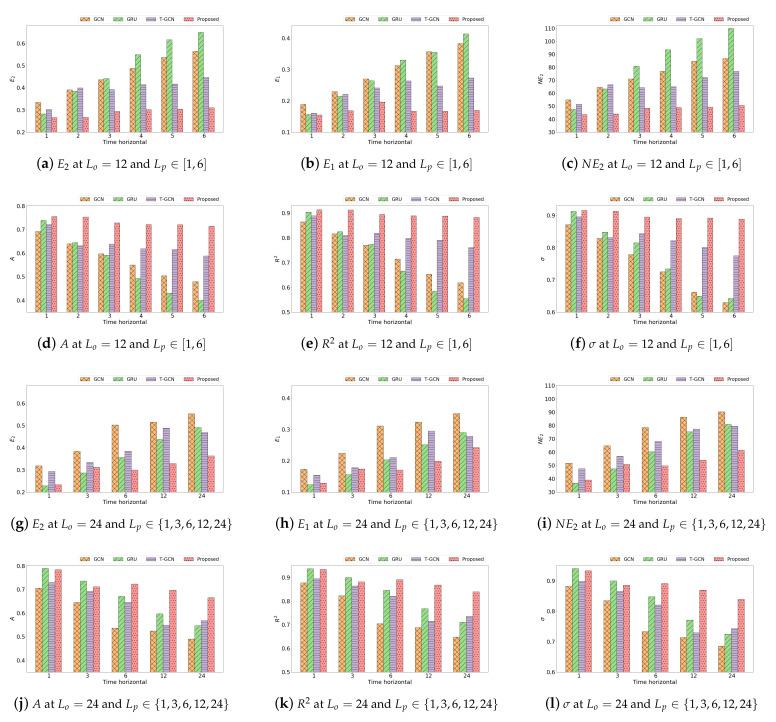
The performance comparison of the proposed model with the existing models according to changes in the prediction and observation periods. X-axes of the plots indicate the prediction sequence length, and Y-axes correspond to the evaluation metrics. $L_{p}$ and $L_{o}$ denote the prediction and observation sequence lengths, respectively.

**Figure A4:**
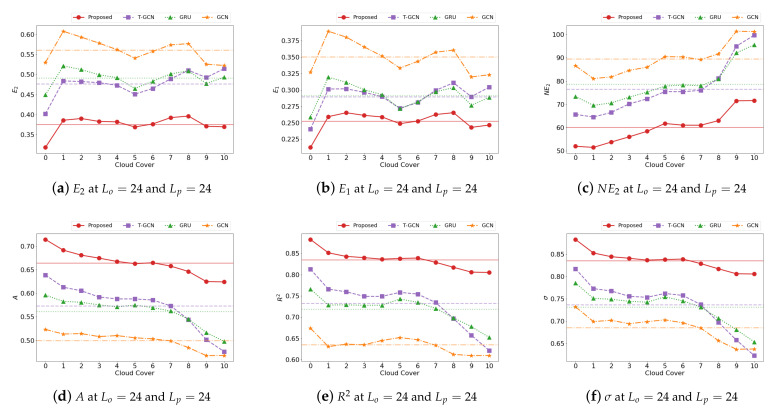
A comparison of the performance stability of the proposed model with the existing models according to the cloud cover. X-axesof the plots indicate the cloudiness level, and Y-axes correspond to the evaluation metrics. The translucent horizontal lines indicate average performance of the models.

The proposed model exhibited the highest accuracy for all cloudiness levels. A performance decrement on cloudy days was commonly observed in all models. However, the decrease in the proposed model was not as severe as that of T-GCN and GRU. Although on a few metrics, the GCN had a similar or lower standard deviation compared to the proposed model, there was a significant difference between the accuracies of the two models. As discussed, the solar irradiance on clear days follows periodic patterns (e.g., daily and yearly). Cloudy days were far less frequent than clear days, as shown in Figure A2. Thus, the high performance of the proposed model supports that the model could predict cloudiness levels the next day by overcoming data imbalance and not merely applying periodic patterns to observations.

In the previous experiment, the T-GCN outperformed the GRU for long-term prediction, whereas the opposite was true for short-term prediction. Similar relationships were observed in this study. Although T-GCN outperformed GRU on clear and slightly cloudy days, GRU performed better than T-GCN on extremely cloudy days (CC ≥8) in most metrics. Even in the CC =10 case, the performance of the T-GCN was similar to or worse than that of the GCN, which focused only on spatial features. For the previous results, we assumed that the combination of spatial and temporal features was effective for understanding meteorological contexts in the long term (RQ1 and RQ2). However, in this experiment, the same combination hindered the recognition of the overcast days. Compared to the proposed model, we can assume that considering solar irradiance in adjacent areas could not provide sufficiently deep contexts to the models, which is different from analyzing spatiotemporal correlations between various meteorological variables (RQ3). Because T-GCN and the proposed model have almost the same architecture, this result would not be achieved from model capabilities for handling the imbalanced distribution of cloudiness. Additionally, the forecasting models commonly showed a rapid performance decrement from CC =7, although they exhibited a relatively stable performance on 2≤ CC ≤6. Partly cloudy skies may not significantly influence the solar irradiance.

#### 4.3.2. Performance Variation according to Months

**Figure A5:**
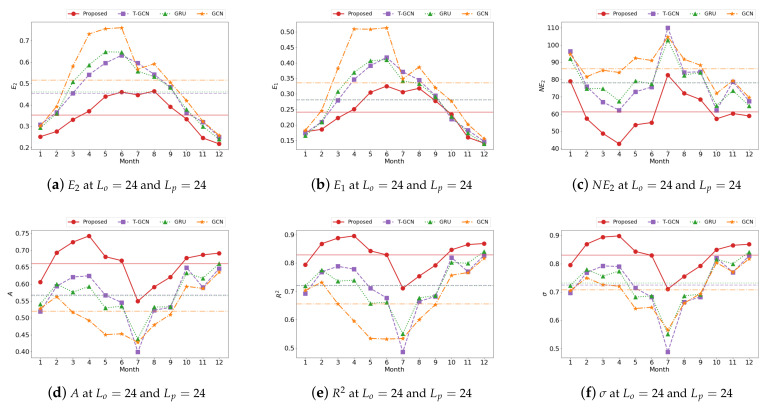
A comparison of the performance stability of the proposed model with the existing models according to the month. X-axes of the plots indicate the month, and Y-axes correspond to the evaluation metrics. The translucent horizontal lines indicate average performance of the models.

The weather on the Korean Peninsula, which is our experimental subject, has four distinct seasons. According to seasonal changes, the weather in each month might have distinctive patterns. We can examine whether the yearly patterns affect the solar irradiance prediction by assessing the forecasting monthly model performance. In addition, the monthly performance can establish the model that can learn yearly patterns or overcome seasonal differences. As in the previous experiment, we segmented our observation samples into months, and the proposed and existing forecasting models were evaluated for each month. Table 5 and Figure A5 list the experimental results.

The proposed model outperformed existing models in most months and metrics. T-GCN and GRU exhibit lower $E_{1}$ values than the proposed model in January and October. The proposed model had a thin lead for the other metrics; we can also see this result from October to December. Considering the previous experiments, T-GCN and GRU exhibited significant performance decrement on cloudy days (Section 4.3.1) and long-term predictions (Section 4.2). Thus, this result could be due to the weather in the Korean Peninsula during winter. (The climate of the Korean Peninsula can be explained as a humid continental climate with humid summers and dry winters. The temperature differences between the hottest part of the summer and the coldest part of the winter are extreme. In addition, most precipitation falls during the summer monsoon period between June and September.) Periodic meteorological patterns were more effective for solar irradiance prediction during winter than the other features. We can also see this point from the result for July, which occurs in the middle of the ‘humid summer’ on the Korean Peninsula. In July, the proposed model had a relatively thin lead for $E_{1}$ but significantly outperformed the other temporal models for normalized accuracy metrics (e.g., *A*, $R^{2}$, and σ). The similar $E_{1}$ values could be caused by the fact that rainy days have low solar irradiance. Thus, although T-GCN and GRU could infer rainy days by analyzing the solar irradiance of adjacent samples, these models failed to predict the solar irradiance on rainy days. Both spatiotemporal and temporal analyses of solar irradiance are insufficient in forecasting nonperiodic and complicated meteorological phenomena. In July, spatiotemporal analysis (T-GCN) underperformed cases in which spatial and temporal features were solely used (GCN and GRU). As discussed for the experiment on cloudiness, considering spatial correlations of solar irradiance only could not make the models understand the meteorological contexts sufficiently deep. However, the proposed model improved this point by analyzing the correlations between the meteorological variables (RQ3).

The T-GCN, GRU, and proposed model exhibited similar tendencies. These models exhibited high normalized accuracy metrics (e.g., $NE_{2}$, *A*, $R^{2}$, and σ) from February to April and October to December, and low accuracy from January and July to September. However, contrary to these models, the performance of the GCN was worse than its average in spring (March to April). Because the spring climate of Korea is dry and has clear characteristics (https://web.kma.go.kr/eng/biz/climate_01.jsp (accessed on 15 August 2022)), this difference is not due to irregular meteorological phenomena, such as precipitation. This result indicates that temporal features are effective for discovering yearly climate patterns.

### 4.4. Parameter Sensitivity Analysis

We assumed that meteorological parameters observed in spatially adjacent areas could influence each other’s future meteorological parameters. For example, the wind speed and direction are affected by the atmospheric pressures of adjacent areas. Therefore, we conducted a temporal analysis of meteorological variables in adjacent areas using the spatiotemporal GCN model. However, there are problems in determining (i) spatially adjacent areas and (ii) correlated meteorological parameters. This section evaluates the effectiveness of the proposed methods for defining spatial adjacency and composing a set of input variables. In addition, we assessed the sensitivity of the proposed model to changes in these two factors.

#### 4.4.1. Meteorological Variable Compositions

The proposed model significantly outperformed the T-GCN [62] by analyzing the spatial correlations between various meteorological variables, and solar irradiance. The previous experiments used all the variables that we collected (listed in Table 1). However, variables that are not correlated with the solar irradiance can hinder the forecasting performance of the proposed model. In addition, if the proposed model can exhibit similar or better performance with fewer input variables than with every variable, the practicality of the model will be improved.

Table 1 presents the Pearson correlation coefficients of solar irradiance with other meteorological variables. We composed the input variable group using a threshold for the correlation coefficient based on the assumption that variables with higher correlations contribute more to forecasting performance. Subsequently, we validate this assumption by adjusting the threshold, as shown in Table 6.

We assume that not all meteorological variables contribute to the forecasting performance of the proposed model. Variables that are less correlated with solar irradiance provide unnecessary and overabundant information for the forecasting model. In addition, if we choose variables that are too strict (i.e., small $\theta_{V}$), the model cannot obtain enough information for understanding weather conditions. Therefore, we expect the model performance to exhibit a convex shape for $\theta_{V}$. Nevertheless, unexpectedly, the proposed model had the best performance when we used all 15 variables ($\theta_{V}=0.00$), with a significant gap. After removing year (YOY) and day (DOY) from 15 ($\theta_{V}=0.04$), the model performance showed a sharp decrease. Subsequently, when we removed precipitation ($P_{t}$) and local pressure ($P_{L}$) from the remaining 13 variables ($\theta_{V}=0.08$), the model exhibited the second highest performance. In addition, this case’s performance was similar to that of the $\theta_{V}=0.50$ case, which used only sunshine duration (*S*) and solar irradiance itself ($S_{r}$). It is not easy to expect the proposed model to understand meteorological phenomena and their spatiotemporal correlations from the two variables. Accordingly, year and day could be key factors for discovering correlations between the variables, despite their low PCC. Therefore, we concluded that the meteorological variables might have non-linear correlations with solar irradiance, and PCC was not sufficient to reflect these correlations. Likewise, the meteorological variables, excluding sunshine duration, air temperature, and relative humidity, have very low PCC with solar irradiance, as shown in Table 1. However, it is difficult to empirically determine the optimal composition of the meteorological variables. Future research will focus on feature selection methods for meteorological variables by combining statistical correlations and domain knowledge in meteorology studies.

#### 4.4.2. The Number of Neighboring Stations

By comparing the ASOS station locations (Figure 1) with the correlation between their historical solar irradiance (Figure A1), we can see that the stations have higher correlations with the closer stations, but the clusters of correlated stations have various sizes. In addition, every observation station had a high PCC (>0.75), and this result indicates that solar irradiance at the ASOS stations has similar long-term tendencies. However, we assume that it is difficult to reflect short-term (hourly or daily) changes and differences in the solar irradiance. Thus, this study employed fixed-size distance-based nearest neighborhoods, which is different from existing studies [43,63] that used flexible-size correlation-based neighborhoods. To examine the advantages and disadvantages of both methods, we assessed the performance fluctuations of the proposed model by adjusting the meteorological networks using both static and dynamic neighborhood sizes, as shown in Table 7.

When we fixed the number of neighborhoods (*N*), the proposed model exhibited the best performance for N=2. Then, the performance worsened according to the increment in neighborhood sizes, and suddenly, the model exhibited the second-highest performance at N=6. After N=6, the model performance deteriorated again with the neighborhood extension. The proposed model assumes that the weather conditions of neighboring stations influence each other. This result indicates that closer stations do not always have a greater influence on future weather conditions. Because observation stations have different geographical features and cannot be located at equal distances, we should search for the optimal number of neighborhoods according to the compositions of the observation stations. In addition, the number of influential stations is not fixed. Thus, we assessed the performance of the proposed model in cases in which we defined the adjacency of the stations according to correlations of their solar irradiance history.

As shown in Figure A1, the observation stations exhibit high PCC values. This could mean that the solar irradiance at most stations exhibited similar tendencies. However, to handle the output instability of solar power plants, regional differences in solar irradiance should be accurately forecast. Although the threshold-based approach could not outperform the nearest neighborhoods, the $\theta_{R}=0.95$ case had the second-highest performance among all cases. However, the $\theta_{R}=0.93$ case underperformed at N=6, and the $\theta_{R}=0.94$ case exhibited a similar performance to the N=9, which is the worst. Considering the values of $\theta_{R}$ in these cases, the performance of the proposed model is sensitive to $\theta_{R}$. Furthermore, if PCC can reflect the meteorological influence between observation points, model performance will show consistent tendencies according to $\theta_{R}$. When $\theta_{R}$ is lower than a certain value, overabundant information can be provided to the model. In addition, with too high $\theta_{R}$, the model cannot obtain sufficient information to analyze the spatial correlations of meteorological features. However, unexpectedly, the performance of the proposed model had an irregular tendency according to $\theta_{R}$, and among the three cases ($\theta_{R}=0.93$, 0.94, and 0.95), $\theta_{R}=0.94$ exhibited a significantly worse performance than the others. This result contradicts the observations above.

Both the distance-based and correlation-based approaches exhibited irregular tendencies. In addition, although the distance-based approach outperformed the correlation-based approach, the difference was not significant. In conclusion, neither approach was sufficient in reflecting the spatial correlations and meteorological influences between the observation areas. Future research should focus on developing measurements of spatial correlations.

## 5. Conclusions

This study aims to conduct day-ahead hourly forecasting of solar irradiance by analyzing the spatio-temporal correlations of solar irradiance with multiple meteorological variables. We also evaluated the effectiveness of (i) spatial analysis, (ii) temporal analysis, and (iii) multivariate analysis for solar irradiance forecasting and validated the underlying research questions presented in Section 1. We collected solar irradiance and other meteorological variables (Table 1) from 42 ASOS stations on the Korean Peninsula (Figure 1). For spatiotemporal analysis of the variables, we modeled the ASOS observation data as a dynamic attribute network, which has the stations as nodes, variables as attributes, and spatial adjacency between the stations as edges. We then developed a novel solar irradiance forecasting model that analyzes the dynamic attributed network and predicts hourly solar irradiance at each station by modifying the AST-GCN model [51].

We evaluated the effectiveness of the proposed model by comparing its prediction accuracy with those of existing deep learning-empowered models and conventional regression models. Subsequently, to validate the practicality of the proposed model, we examined its accuracy according to the prediction sequence lengths (from hour-ahead to day-ahead prediction), cloudiness, months, variable compositions, and edge density of the network. The proposed model outperformed the existing models, especially in terms of long-term prediction. Contrarily, most of the existing studies have been limited in intra-day prediction (1 to 6 h ahead) [18]. The comparison between the performances of the proposed and existing models indicates that the spatial, temporal, and multivariate features of atmospheric data are synergistic for predicting solar irradiance. Although a few previous studies [43,46,50] attempted to combine temporal and spatial features or used both temporal and multivariate features, there have barely been either forecasting models integrating the three factors or validations for synergistic effects among the factors. In addition, the proposed model exhibited higher and more stable performances on most cloudiness levels and months than the existing models. The proposed model exhibited performance decrements on overcast days and summer as with the existing ones. However, more or less, solar irradiance forecasting models are difficult to avoid this problem caused by frequent thunderstorms in the summer of the Korean Peninsula [75,76]. The experiment for variable compositions showed that the correlation coefficients were insufficient in reflecting spatiotemporal correlations between meteorological variables. Likewise, both geographic distances and correlation coefficients were insufficient in establishing spatial influences between the atmospheric contexts of the observatories. In future research, we will address the following limitations:

- Prediction sequence length: We evaluated the forecasting performance of the proposed and existing models on multiple prediction sequence lengths (from an hour-ahead to a day-ahead prediction). However, predicting solar irradiance with longer time intervals (e.g., a week or a month) will be helpful for the practical usage of solar power. We assume that the long-term dependency problem caused by adopting GRU layers hindered the long-term prediction performance of the proposed model. In further research, we will improve this problem by applying the attention mechanism to consider relative importance of time points, adjacent stations, and meteorological variables.
- Low accuracy on high cloud cover: The proposed model showed performance decrement on cloudy days, although the decrement was not as significant as the existing models. This problem might come from difficulties in predicting solar irradiance on cloudy days but also due to forecasting cloudiness. Wind speeds and directions at high altitudes are closely correlated with cloudiness [77], and future research will attempt to consider these variables in addition to those observed at the ground observatories.
- Multi-modal analysis: Atmospheric observation data are collected through various devices (e.g., sensors, radars, cameras, etc.) deployed on ground stations, satellites, observation balloons, aircraft, etc. Despite the variety of observation data, this study has focused on sensor data from ground observatories. Combining the multi-modal and multi-aspect observations will enable forecasting models to discover more accurate information for atmospheric contexts. For example, the ground observatories were not located with a uniform gap, and geographical characteristics in the gaps were also not homogeneous. Thus, covering the gaps by incorporating geographical features [38,78], land usages [79], and satellite data [80] will be effective for analyzing spatial correlations between atmospheric contexts of the observatories.

## Figures and Tables

**Table 1:** Meteorological variables associated with ASOS data for solar irradiance prediction. The fourth column presents absolute values of Pearson correlation coefficients of historical solar irradiance (01/2017–12/2019) with other meteorological variables.

Type	Variables	Notation	Units	Corr.
Geographical Parameter	Latitude	$L_{a}$	${}^{\circ }$	-
	Longitude	$L_{o}$	${}^{\circ }$	-
Calendar Parameter	The year	YOY	-	0.00
	The day of the year	DOY	-	0.00
	The month of the year	MOY	-	0.05
	The hour of the day	HOD	-	0.16
Meteorological Parameter	Sunshine duration	*S*	h	0.82
	Air temperature	$T_{a}$	${}^{\circ }$C	0.31
	Relative humidity	RH	%	0.48
	Local pressure	$P_{L}$	hPa	0.04
	Sea level pressure	$P_{s}$	hPa	0.09
	Precipitation	$P_{t}$	mm	0.07
	Cloud cover	CC	-	0.23
	Wind speed	$W_{s}$	ms${}^{-1}$	0.20
	Wind direction	$W_{d}$	-	0.14
	Visibility	VIS	m	0.18
Forecasting Target	Solar irradiance	$S_{r}$	MJm${}^{-2}$	1.00

**Table 2:** A performance comparison of the proposed model with the baseline methods. The upper, middle, and lower parts of this table present the accuracy of the conventional regression models, the deep learning-empowered models, and the proposed model, respectively. Furthermore, the left and right sides exhibit the model accuracy of the univariate and multivariate analysis, respectively. - indicates cases that we cannot assess the models’ accuracy due to their characteristics. Additionally, * and ** denote models with the first and second best performance on each case and metric, respectively. The hyper-parameters were set as $L_{o}=12$, $L_{p}=1$, N=4, and $\theta_{V}=0.00$.

	Univariate Analysis						Multivariate Analysis					
	$E_{2}$	$E_{1}$	${\mathrm{NE}}_{2}$	A	$R^{2}$	σ	$E_{2}$	$E_{1}$	${\mathrm{NE}}_{2}$	A	$R^{2}$	σ
HA	1.03	0.78	173.02	0.05	−0.29	−0.29	-	-	-	-	-	-
ARIMA	0.87	0.72	152.16	0.16	−0.01	0.00	-	-	-	-	-	-
VAR	-	-	-	-	-	-	0.91	0.70	155.81	0.16	−0.01	0.00
SVR	0.27 *	0.17	44.91 *	0.74 **	0.90 **	0.91 **	0.92	0.68	192.08	0.10	0.00	0.00
MLP	0.34	0.22	56.16	0.32	0.86	0.86	0.86	0.67	145.39	0.20	0.09	0.09
GCN	0.33	0.19	55.82	0.69	0.86	0.87	0.43	0.27	72.63	0.60	0.77	0.78
GRU	0.28 **	0.16 *	47.48 **	0.74 *	0.90 *	0.91 *	0.45	0.27	75.55	0.58	0.76	0.79
T-GCN	0.30	0.16 **	53.41	0.72	0.89	0.90	0.33 **	0.18 **	53.80 **	0.70 **	0.87 **	0.89 **
**Proposed**	-	-	-	-	-	-	0.23 *	0.12 *	38.07 *	0.79 *	0.94 *	0.94 *

**Table 3:** The performance of the proposed and existing models according to the prediction and observation sequence lengths. The left and right sides of this table exhibit the model accuracy on cases that the observation sequence length is 12 and 24, respectively. Subsequently, each column presents changes in the model accuracy according to the prediction sequence length. Furthermore, * and ** denote models with the first and second best performance on each case and metric, respectively. The remaining hyper-parameters were set as N=4 and $\theta_{V}=0.00$.

Observation		12						24				
**Prediction**		1	2	3	4	5	6	1	3	6	12	24
$E_{2}$	MLP	0.86	0.88	0.90	0.91	0.90	0.91	0.86	0.86	0.86	0.86	0.88
	GCN	0.33	0.39	0.44	0.49	0.54	0.56	0.32	0.38	0.50	0.52	0.55
	GRU	0.28 **	0.38 **	0.44	0.55	0.62	0.65	0.23 *	0.29 *	0.36 **	0.44 **	0.49
	T-GCN	0.30	0.40	0.39 **	0.41 **	0.42 **	0.45 **	0.29	0.33	0.38	0.49	0.47 **
	**Proposed**	0.27 *	0.27 *	0.29 *	0.30 *	0.30 *	0.31 *	0.23 **	0.31 **	0.30 *	0.33 *	0.36 *
$E_{1}$	MLP	0.67	0.69	0.71	0.72	0.71	0.72	0.67	0.67	0.66	0.66	0.69
	GCN	0.19	0.23	0.27	0.31	0.36	0.38	0.17	0.22	0.31	0.32	0.35
	GRU	0.16 **	0.21 **	0.26	0.33	0.36	0.41	0.12 *	0.16 *	0.20 **	0.25 **	0.29
	T-GCN	0.16	0.22	0.24 **	0.26 **	0.25 **	0.27 **	0.15	0.18	0.21	0.30	0.28 **
	**Proposed**	0.15 *	0.17 *	0.20 *	0.17 *	0.17 *	0.17 *	0.13 **	0.17 **	0.17 *	0.20 *	0.24 *
$NE_{2}$	MLP	145.39	147.29	150.92	152.43	152.19	152.42	144.93	144.90	146.06	151.65	152.49
	GCN	54.93	64.55	71.10	76.99	84.67	86.77	51.78	64.90	78.56	86.41	90.33
	GRU	47.49 **	63.29 **	80.82	93.47	102.16	109.91	36.77 *	47.54 *	60.39 **	75.26 **	80.93
	T-GCN	51.57	66.56	64.45 **	64.97 **	72.08 **	76.88 **	47.69	56.90	68.17	77.46	79.65 **
	**Proposed**	43.55 *	44.05 *	48.53 *	49.03 *	49.11 *	50.60 *	39.11 **	50.74 **	49.72 *	54.01 *	61.29 *
*A*	MLP	0.20	0.19	0.17	0.16	0.16	0.16	0.21	0.20	0.21	0.20	0.19
	GCN	0.69	0.64	0.60	0.55	0.50	0.48	0.71	0.65	0.54	0.52	0.49
	GRU	0.74 **	0.64 **	0.59	0.49	0.43	0.40	0.79 *	0.74 *	0.67 **	0.60 **	0.55
	T-GCN	0.72	0.63	0.64 **	0.62 **	0.62 **	0.59 **	0.73	0.69	0.65	0.55	0.57 **
	**Proposed**	0.75 *	0.75 *	0.73 *	0.72 *	0.72 *	0.71 *	0.78 **	0.71 **	0.72 *	0.70 *	0.66 *
$R^{2}$	MLP	0.09	0.06	0.01	0.00	0.00	0.00	0.09	0.09	0.10	0.09	0.04
	GCN	0.86	0.82	0.77	0.71	0.65	0.62	0.88	0.82	0.70	0.69	0.65
	GRU	0.90 **	0.82 **	0.77	0.67	0.58	0.55	0.94 *	0.90 *	0.85 **	0.77 **	0.71
	T-GCN	0.89	0.81	0.82 **	0.80 **	0.79 **	0.76 **	0.90	0.86	0.82	0.72	0.74 **
	**Proposed**	0.91 *	0.91 *	0.89 *	0.89 *	0.89 *	0.88 *	0.93 **	0.88 **	0.89 *	0.87 *	0.84 *
σ	MLP	0.09	0.06	0.02	0.00	0.00	0.00	0.09	0.09	0.11	0.09	0.05
	GCN	0.87	0.83	0.78	0.73	0.66	0.63	0.88	0.84	0.73	0.71	0.69
	GRU	0.91 **	0.85 **	0.81	0.73	0.65	0.64	0.94 *	0.90 *	0.85 **	0.77 **	0.72
	T-GCN	0.90	0.83	0.84 **	0.82 **	0.80 **	0.77 **	0.90	0.87	0.82	0.73	0.74 **
	**Proposed**	0.92 *	0.91 *	0.89 *	0.89 *	0.89 *	0.89 *	0.93 **	0.89 **	0.89 *	0.87 *	0.84 *

**Table 4:** Performance of the proposed and existing models according to cloud cover. The first row indicates the degree of cloud cover from 0 to 10. The second row presents the distribution of cloudiness levels in the experimental dataset. In the third to thirteenth columns, * and ** indicate cloudiness levels that each forecasting model had the best and second-best performances, respectively. Additionally, in the last two columns, * and ** refer to forecasting models that showed the best and second-best performances on average and the lowest and second-lowest standard deviation, respectively. The hyper-parameters were set as $L_{o}=24$, $L_{p}=24$, N=4, and $\theta_{V}=0.00$.

		Cloud Cover											Statistics	
		0	1	2	3	4	5	6	7	8	9	10	Avg.	S.D.
**Ratio (%)**		**25.14**	3.63	3.60	3.91	3.94	4.58	6.29	7.59	8.85	11.01	21.44	-	-
$E_{2}$	GCN	0.53	0.61	0.59	0.58	0.56	0.54	0.56	0.57	0.58	0.53 **	0.52 *	0.56	0.03
	GRU	0.45 *	0.52	0.51	0.50	0.49	0.47 **	0.48	0.50	0.51	0.48	0.49	0.49	0.02 **
	T-GCN	0.40 *	0.48	0.48	0.48	0.47	0.45 **	0.47	0.49	0.51	0.49	0.51	0.48 **	0.03
	**Proposed**	0.32 *	0.39	0.39	0.38	0.38	0.37	0.38	0.39	0.40	0.37	0.37 **	0.38 *	0.02 *
$E_{1}$	GCN	0.33	0.39	0.38	0.37	0.35	0.33	0.34	0.36	0.36	0.32 *	0.32 **	0.35	0.02
	GRU	0.26 *	0.32	0.31	0.30	0.29	0.27 **	0.28	0.30	0.30	0.28	0.29	0.29	0.02
	T-GCN	0.24 *	0.30	0.30	0.30	0.29	0.27 **	0.28	0.30	0.31	0.29	0.30	0.29 **	0.02 **
	**Proposed**	0.21 *	0.26	0.27	0.26	0.26	0.25	0.25	0.26	0.27	0.24 **	0.25	0.25 *	0.02 *
$NE_{2}$	GCN	86.60	81.10 *	81.80 **	84.63	85.99	90.53	90.43	89.27	91.73	101.46	101.35	89.54	6.50 *
	GRU	73.37	69.55 *	70.62 **	73.06	75.27	77.84	78.34	77.98	80.94	92.15	95.66	78.62	7.95
	T-GCN	65.64 **	64.55 *	66.50	70.18	72.33	75.45	75.42	76.02	81.15	95.04	99.80	76.55 **	10.99
	**Proposed**	51.93 *	51.45 **	53.76	56.00	58.38	61.75	61.03	60.99	62.98	71.52	71.62	60.13 *	6.59 **
*A*	GCN	0.52 *	0.51	0.51 **	0.51	0.51	0.51	0.50	0.50	0.48	0.47	0.47	0.50	0.02 *
	GRU	0.60 *	0.58 **	0.58	0.58	0.57	0.58	0.57	0.56	0.55	0.52	0.50	0.56	0.03
	T-GCN	0.64 *	0.61 **	0.61	0.59	0.59	0.59	0.59	0.57	0.54	0.50	0.48	0.57 **	0.05
	**Proposed**	0.71 *	0.69 **	0.68	0.67	0.67	0.66	0.66	0.66	0.65	0.62	0.62	0.66 *	0.03 **
$R^{2}$	GCN	0.67 *	0.63	0.64	0.63	0.65	0.65 **	0.65	0.63	0.61	0.61	0.61	0.64	0.02 **
	GRU	0.77 *	0.73	0.73	0.73	0.73	0.74 **	0.73	0.72	0.70	0.68	0.65	0.72	0.03
	T-GCN	0.81 *	0.77 **	0.76	0.75	0.75	0.76	0.75	0.73	0.70	0.66	0.62	0.73 **	0.05
	**Proposed**	0.88 *	0.85 **	0.84	0.84	0.84	0.84	0.84	0.83	0.82	0.81	0.81	0.84 *	0.02 *
σ	GCN	0.73 *	0.70	0.70	0.69	0.70	0.70 **	0.70	0.68	0.66	0.64	0.64	0.69	0.03 **
	GRU	0.79 *	0.75	0.75	0.74	0.74	0.75 **	0.75	0.73	0.71	0.68	0.65	0.73	0.04
	T-GCN	0.82 *	0.77 **	0.77	0.76	0.75	0.76	0.76	0.74	0.70	0.66	0.62	0.74 **	0.06
	**Proposed**	0.88 *	0.85 **	0.84	0.84	0.84	0.84	0.84	0.83	0.82	0.81	0.81	0.84 *	0.02 *

**Table 5:** Performance of the proposed and existing models according to months. In the third to fourteenth columns, * and ** indicate months when each forecasting model had the best and second-best performances, respectively. Additionally, in the last two columns, * and ** denote forecasting models that showed the best and second-best performances on average and the lowest and second-lowest standard deviation, respectively. The hyper-parameters were set as $L_{o}=24$, $L_{p}=24$, N=4, and $\theta_{V}=0.00$.

		Months												Statistics	
		1	2	3	4	5	6	7	8	9	10	11	12	Avg.	S.D.
$E_{2}$	GCN	0.30 **	0.39	0.58	0.73	0.76	0.76	0.57	0.59	0.50	0.42	0.32	0.26 *	0.52	0.18
	GRU	0.29 **	0.36	0.51	0.59	0.65	0.65	0.56	0.53	0.48	0.38	0.30	0.24 *	0.46	0.14
	T-GCN	0.31 **	0.37	0.45	0.54	0.60	0.63	0.60	0.54	0.48	0.36	0.32	0.25 *	0.45 **	0.13 **
	**Proposed**	0.25 **	0.28	0.33	0.37	0.44	0.46	0.45	0.46	0.39	0.33	0.25	0.22 *	0.35 *	0.09 *
$E_{1}$	GCN	0.18 **	0.25	0.38	0.51	0.51	0.51	0.35	0.39	0.32	0.28	0.20	0.16 *	0.34	0.13
	GRU	0.17 **	0.21	0.31	0.37	0.41	0.41	0.34	0.33	0.29	0.23	0.17	0.14 *	0.28 **	0.10
	T-GCN	0.17 **	0.21	0.28	0.35	0.39	0.42	0.37	0.34	0.29	0.22	0.18	0.15 *	0.28	0.09 **
	**Proposed**	0.18	0.19	0.22	0.25	0.30	0.33	0.31	0.32	0.28	0.23	0.16 **	0.14 *	0.24 *	0.07 *
$NE_{2}$	GCN	94.69	81.64	85.37	84.08	92.46	91.04	104.82	91.73	88.30	71.97 **	79.33	69.56 *	85.48	9.49 *
	GRU	92.04	74.71	74.77	67.44	79.23	77.41	102.95	82.48	84.15	64.91 **	73.57	64.76 *	76.94	10.31 **
	T-GCN	96.46	75.91	66.93	62.22	72.84	75.61	110.04	84.11	84.51	62.13 *	78.73	67.45 **	76.41 **	12.97
	**Proposed**	78.96	57.30	48.71 **	42.67 *	53.67	55.03	82.55	72.02	68.38	57.19	60.25	58.85	59.69 *	10.58
*A*	GCN	0.53	0.56	0.52	0.49	0.45	0.45	0.43	0.48	0.51	0.59 **	0.59	0.64 *	0.52	0.06
	GRU	0.54	0.60	0.58	0.59	0.53	0.53	0.44	0.53	0.53	0.63 **	0.62	0.66 *	0.57	0.06 **
	T-GCN	0.52	0.59	0.62	0.62	0.57	0.55	0.40	0.52	0.53	0.65 *	0.59	0.65 **	0.57 **	0.07
	**Proposed**	0.61	0.69	0.72 **	0.74 *	0.68	0.67	0.55	0.59	0.62	0.68	0.69	0.69	0.66 *	0.06 *
$R^{2}$	GCN	0.70	0.73	0.66	0.60	0.53	0.53	0.53	0.60	0.65	0.76	0.77 **	0.82 *	0.66	0.10
	GRU	0.72	0.78	0.74	0.74	0.66	0.66	0.55	0.68	0.69	0.80 **	0.80	0.84 *	0.72	0.08 **
	T-GCN	0.69	0.77	0.79	0.78	0.71	0.68	0.49	0.66	0.68	0.82 **	0.77	0.83 *	0.72 **	0.09
	**Proposed**	0.79	0.87	0.89 **	0.90 *	0.84	0.83	0.71	0.75	0.79	0.85	0.87	0.87	0.83 *	0.06 *
σ	GCN	0.71	0.75	0.73	0.72	0.64	0.65	0.57	0.66	0.69	0.80 **	0.77	0.82 *	0.71	0.07 **
	GRU	0.72	0.78	0.76	0.77	0.68	0.69	0.55	0.69	0.69	0.82 **	0.80	0.84 *	0.73 **	0.08
	T-GCN	0.70	0.77	0.79	0.79	0.71	0.68	0.49	0.67	0.68	0.82 **	0.77	0.83 *	0.72	0.09
	**Proposed**	0.80	0.87	0.89 **	0.90 *	0.84	0.83	0.71	0.76	0.79	0.85	0.87	0.87	0.83 *	0.06 *

**Table 6:** Performance of the proposed model according to the variable composition. The first row presents the thresholds of Pearson correlation coefficients of the forecasting target (i.e., solar irradiance) with the other meteorological variables ($\theta_{V}$). The second row shows the number of variables chosen based on the thresholds. Additionally, * and ** indicate that the proposed model had the best and second-best performances with the corresponding $\theta_{V}$, respectively. The remaining hyper-parameters were set as $L_{o}=12$, $L_{p}=1$, and N=4.

Corr. Threshold ($\theta_{V}$)	0.50	0.40	0.30	0.20	0.10	0.08	0.04	0.00
# Variables	2	3	4	6	9	11	13	15
$E_{2}$	0.33 **	0.37	0.40	0.40	0.42	0.33	0.37	0.27 *
$E_{1}$	0.18 **	0.20	0.22	0.22	0.22	0.19	0.23	0.15 *
$NE_{2}$	54.62 **	62.00	67.26	66.66	70.75	54.77	61.53	43.55 *
*A*	0.70 **	0.65	0.63	0.63	0.61	0.69	0.66	0.75 *
$R^{2}$	0.87 **	0.83	0.80	0.80	0.78	0.87	0.84	0.91 *
σ	0.89 **	0.86	0.84	0.84	0.82	0.88	0.84	0.92 *

**Table 7:** Performance of the proposed model according to the edge density of meteorological networks. The edge density was adjusted using the number of neighborhood stations (*N*) and the threshold for the Pearson correlation coefficient between historical solar irradiance of stations ($\theta_{R}$). # Nodes and # Edges indicate the number of nodes and edges, respectively, in the meteorological networks constructed according to *N* and $\theta_{R}$. In addition, * and ** denote that the proposed model had the best and second-best performances with the corresponding *N* and $\theta_{R}$, respectively. The remaining hyper-parameters were set as $L_{o}=12$, $L_{p}=1$, and $\theta_{V}=0.00$.

	# Neighborhoods (*N*)									Corr. Threshold ($\theta_{R}$)		
	1	2	3	4	5	6	7	8	9	0.93	0.94	0.95
#**Nodes**	**42**	**42**	**42**	**42**	**42**	**42**	**42**	**42**	**42**	**42**	**42**	**42**
#**Edges**	**27**	**53**	**80**	**104**	**129**	**153**	**181**	**204**	**229**	**137**	**85**	**46**
$E_{2}$	0.28	0.23 *	0.26	0.27	0.29	0.24 **	0.29	0.29	0.30	0.25 **	0.29	0.24 *
$E_{1}$	0.18	0.12 *	0.17	0.15	0.18	0.13 **	0.18	0.16	0.18	0.13 **	0.18	0.13 *
$NE_{2}$	45.87	38.07 *	43.06	43.55	48.52	39.65 **	47.45	48.19	50.79	42.08 **	49.33	40.79 *
*A*	0.74	0.79 *	0.76	0.75	0.73	0.78 **	0.73	0.73	0.72	0.77 **	0.73	0.78 *
$R^{2}$	0.90	0.94 *	0.92	0.91	0.90	0.93 **	0.90	0.90	0.89	0.92 **	0.90	0.93 *
σ	0.91	0.94 *	0.92	0.92	0.90	0.93 **	0.90	0.90	0.89	0.93 **	0.90	0.93 *

## Data Availability

The meteorological data used in this study are openly available in Open MET Data Portal (https://data.kma.go.kr/cmmn/main.do (accessed on 15 August 2022) operated by KMA (Korea Meteorological Administration).

## Appendix A

### Appendix A.1. Data Statistics

This section presents figures and tables that provide the details of our experimental dataset.

### Appendix A.2. Experimental Results

In this section, we visualize our experimental results to enhance readability.
